# Relative contributions of various endogenous and exogenous factors to the mosquito microbiota

**DOI:** 10.1186/s13071-020-04491-7

**Published:** 2020-12-10

**Authors:** Haikel N. Bogale, Matthew V. Cannon, Kalil Keita, Denka Camara, Yaya Barry, Moussa Keita, Drissa Coulibaly, Abdoulaye K. Kone, Ogobara K. Doumbo, Mahamadou A. Thera, Christopher V. Plowe, Mark Travassos, Seth Irish, David Serre

**Affiliations:** 1 Institute for Genome Sciences, University of Maryland School of Medicine, Baltimore, MD USA; 2 Programme National de Lutte contre le Paludisme, Conakry, Guinea; 3 Malaria Research and Training Center, University Science, Techniques and Technologies of Bamako, Bamako, Mali; 4 Duke Global Health Institute, Duke University, Durham, NC USA; 5 Malaria Research Program, Center of Vaccine Development and Global Health, University of Maryland School of Medicine, Baltimore, MD USA; 6 U.S. President’s Malaria Initiative and Entomology Branch, Division of Parasitic Diseases and Malaria, Center for Global Health, US Centers for Disease Prevention, Atlanta, GA USA

**Keywords:** Microbiome, *Anopheles*, High-throughput screening, Bacterial composition, Entomological control

## Abstract

**Background:**

The commensal microbiota of mosquitoes impacts their development, immunity, and competency, and could provide a target for alternative entomological control approaches. However, despite the importance of the mosquito/microbiota interactions, little is known about the relative contribution of endogenous and exogenous factors in shaping the bacterial communities of mosquitoes.

**Methods:**

We used a high-throughput sequencing-based assay to characterize the bacterial composition and diversity of 665 individual field-caught mosquitoes, as well as their species, genotype at an insecticide resistance locus, blood-meal composition, and the eukaryotic parasites and viruses they carry. We then used these data to rigorously estimate the individual effect of each parameter on the bacterial diversity as well as the relative contribution of each parameter to the microbial composition.

**Results:**

Overall, multivariate analyses did not reveal any significant contribution of the mosquito species, insecticide resistance, or blood meal to the bacterial composition of the mosquitoes surveyed, and infection with parasites and viruses only contributed very marginally. The main driver of the bacterial diversity was the location at which each mosquito was collected, which explained roughly 20% of the variance observed.

**Conclusions:**

This analysis shows that when confounding factors are taken into account, the site at which the mosquitoes are collected is the main driver of the bacterial diversity of wild-caught mosquitoes, although further studies will be needed to determine which specific components of the local environment affect bacterial composition.

**Graphical Abstract:**

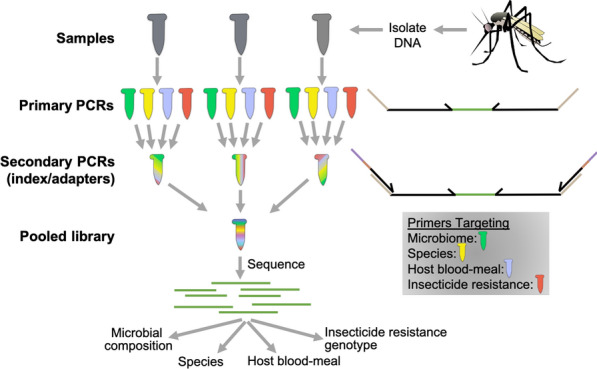

## Background

*Aedes*, *Culex*, and *Anopheles* mosquitoes can transmit eukaryotic parasites and viruses to humans and are responsible for devastating diseases, such as malaria, lymphatic filariasis, dengue, West Nile, and Zika, that affect hundreds of millions of people worldwide and cause hundreds of thousands of deaths annually [1]. Successful disease elimination campaigns have focused on interrupting transmission of these diseases by targeting their mosquito vectors. Thus, in the early twentieth century, vector control approaches primarily relied on the use of chemicals and larviciding tools (i.e., petroleum oils and larvivorous fish) to eliminate larval and adult stages of the mosquito [2, 3]. Environmental and human health concerns brought by the persistent use of chemicals like dichlorodiphenyltrichloroethane (DDT) over the years have partly led to the increased use of pyrethroids, which are safer alternatives, in vector control measures [3]. In the last three decades, entomological control strategies based on pyrethroid-treated bed nets and indoor residual spraying have been extensively used in the fight against vector-borne diseases with considerable success in reducing disease burden [4–6]. However, wide-spread use of these approaches, combined with exposure to agricultural pest control chemicals, have led to the emergence and rapid spread of insecticide resistance alleles in many areas [7, 8]. In addition, several populations of mosquitoes have modified their behaviors (e.g., their host biting time [9, 10] and location [11, 12] or their host species preference [13, 14]) upon exposure to insecticides. This acquisition of chemical and behavioral resistance to insecticides threatens the advances made in control of mosquito-borne diseases and highlights the need for alternative measures.

One alternative to chemicals is to leverage biological agents to control mosquito populations (sometimes referred to as biological controls). For example, *Bacillus thuringiensis*, a spore-forming bacterium with larvicidal characteristics, is used extensively against disease-transmitting insects as well as agricultural pests [15]. In recent years, the use of *Wolbachia* strains as biological control agents has also gained momentum, with studies revealing an ability of these bacteria to promote pathogen interference and to reduce the life span of mosquitoes [16–18]. Furthermore, recent findings have highlighted how modifications of the bacterial communities present in the midgut of mosquitoes could decrease or inhibit transmission of pathogens. For example, studies have demonstrated the importance of gut microbiota in mosquito larval development and shown that bacteria are required for *Aedes* mosquitoes to survive to the adult stages [19, 20]. Similarly, it has been shown that elimination of native microbiota resulted in delayed growth in *Anopheles* larvae [21]. Functional studies on adult stage mosquitoes showed that the gut microbiota can increase the resistance of mosquitoes to human pathogens by modulating the mosquito innate immune response [22, 23] or directly through production of anti-pathogen molecules from specific microbial species [24, 25]. Overall, these studies demonstrate the potential of microbiota manipulations for inhibiting pathogen transmission and/or reducing vector competence.

However, while these laboratory and field studies highlight the role of the mosquito midgut microbiota in regulating the development and transmission of human pathogens, very little is known about the factors that shape the diversity of the bacterial composition in wild mosquitoes. Some studies have suggested that the location of mosquito collection is associated with the composition of the microbiota [26, 27], while others have shown that the microbial composition differed between mosquito species, even when they are closely related and collected at the same location [28], or reared under the same conditions [20]. In addition, it is possible that genetic resistance to insecticides may also influence the microbial composition of field-collected adult mosquitoes (e.g., resistance could result in differential exposure of the gut microbiota to insecticides, which can alter the composition of the microbiota, see, for example, [29]). Lastly, the source of the blood meal has also been shown to impact gut microbiota composition, with the mammalian blood-meal source altering the gut bacterial composition of adult mosquitoes [30]. However, since these studies typically addressed only one of those factors at a time (without correcting for confounding effects), the relative contribution of each of these parameters to shaping the midgut microbiota composition of wild-caught mosquitoes remains unclear.

We report here the results of our analysis of 665 individual *Anopheles* mosquitoes collected in Guinea and Mali. We characterized the microbial composition of these mosquitoes and screened them for a large variety of eukaryotic parasites and viruses. We also characterized the species of all mosquitoes, genotyped them at a major associated insecticide resistance locus, and examined the source of their last blood meal. We then tested how these endogenous and exogenous factors influenced the bacterial diversity and simultaneously estimated the relative contribution of these factors to the mosquito microbiota.

### Methods

### Sample collections

Mosquitoes were collected from six sites in Guinea by human landing catches (HLC) and pyrethrum spray catches (PSC) between August and November 2016 and in August 2017 (Table 1; Additional file 1: Figure S1). The captured mosquitoes were placed in Eppendorf tubes containing ethanol and shipped to the University of Maryland School of Medicine for analysis. Mosquitoes were also collected in Mali from homes in Bandiagara using PSC in July 2011. The captured mosquitoes were placed in Carnoy’s solution (volume ratio acetic acid:ethanol, 1:3) and shipped to the molecular biology laboratory of the Malaria Research and Training Center (MRTC) in Bamako, Mali, for DNA extraction. See Table 1 for details on the collection sites and the collection method used.

**Table 1:** Mosquito collection locations, ecoregions, methods, dates, and numbers

Country	Site	Ecoregion	Collection methods used	Number of mosquitoes	Number of species	Collection dates
Guinea	Kissidougou	Guinean forest savanna mosaic	HLC	118	2	August 2017
	Kankan	West Sudanian savanna	HLC/PSC	178	3	August 2017
	Faranah	Guinean forest savanna mosaic	HLC	79	3	August/September 2016
	Dabola	Guinean montane forest	HLC	79	4	August 2017
	Boffa	Guinean forest savanna mosaic	HLC	89	4	July 2017
	Mamou	Guinean forest savanna mosaic	HLC	41	1	October/November 2016
Mali	Bandiagara	West Sudanian savanna	PSC	81	2	July 2011

HLC, human landing catch; PSC, pyrethrum spray catch

### Guinea mosquito DNA extraction

DNA was extracted from each mosquito using a modified version of the Qiagen 96-well extraction protocol (Qiagen N.V., Hilden, Germany). Briefly, whole individual mosquitoes were randomly placed in each well of a 96-well plate with five 1-mm RNASE free oxide beads for homogenization; 11–14 extraction controls were also included on each plate. Each mosquito was homogenized using a TissueLyser for 6 min at 20 m/s in the lysis buffers provided by the Qiagen DNeasy 96 Blood & Tissue Kit (Qiagen N.V.). The plates were then centrifuged at 1500 *g* for 3 min. The homogenates were then incubated for 1 h at 55 °C, centrifuged again, and incubated overnight at 55 °C. After a final centrifugation step, the supernatant was transferred to a 96-well DNeasy plate to bind the DNA. The columns were washed twice before elution of the DNA with 100 μl of Qiagen buffer AE (Qiagen N.V.). A NanoDrop spectrophotometer (Thermo Fisher Scientific, Waltham, MA, USA) was used to determine DNA concentration.

### Mali mosquito DNA extraction

DNA was extracted from the body section of each mosquito using the Chelex-100 chelating resin (Bio-Rad Laboratories, Hercules, CA, USA) protocol. Briefly, a dissecting needle was used to separate out the thorax and abdomen sections of each individual mosquito, each of which was then placed in 1.5-ml tubes containing deionized water. Pipette tips were used to grind each sample in the tube, following which each sample was further homogenized for 20 min in phosphate buffered saline (PBS; 1×)/1% saponin solution, with gentle shaking. The homogenates were then incubated at room temperature (25 °C) overnight, following which the tubes were centrifuged at 20,000 *g* for 2 min and the supernatants discarded. After the pellets were washed with PBS (1×), each pellet was resuspended in 75 μl of deionized water and 25 μl of 20% Chelex-100 chelating resin solution. This mixture was placed on a heating block for at least 10 min and stirred every 5 min. DNA was transferred into a new tube after a final centrifugation step for 1 min.

### PCR primers

DNA extracted from each mosquito was amplified using primers targeting bacterial 16S rRNA primers for microbiota analysis [31], mosquito knockdown resistance west (*kdr*-west L1014F mutation variant) for insecticide resistance genotyping [32], cytochrome* c* oxidase 1 (COX1) and S200X6.1 [33] loci for mosquito species determination, mammalian mitochondrial 16S rRNA sequences for blood-meal analysis, and eukaryotic parasite and virus primers (targeting 18S rRNA and NS5 loci, respectively) [34] for identification of parasite and virus species (Additional file 2: Table S1). The rationale for targeting these specific loci and the primer design are described in detail in the respective references. Briefly, all primers were designed or selected to simultaneously fulfil the following conditions: (i) allow amplification of all sequences of the taxon targeted, (ii) avoid off-target amplification, (iii) provide sufficient sequence information for resolving the genotype or taxonomy, and (iv) be sufficiently short to be sequenced using Illumina technology and with paired end overlaps to allow for sequencing error-correction.

### DNA amplification

DNA extracted from 665 individual mosquitoes and from the 95 extraction control samples was amplified separately with each primer pair using the Promega GoTaq DNA Polymerase (Promega Corp., Madison, WI, USA) under the following conditions: initial denaturing step at 95 °C for 2  min, followed by 40 cycles of 95 °C for 30 s, 50 °C for 30 s and 72 °C for 30 s, and a final extension of 5 min. Only 35 cycles were used to amplify bacterial 16S rRNA.

### RNA virus detection

Viral sequences were analyzed by first synthesizing cDNA from carry-over RNA present in DNeasy extracted samples using M-MLV Reverse Transcriptase (Promega Corp.). Briefly, 2 µl of mosquito DNA extract from each sample was incubated with 1 µl of random hexamers (0.5 µg) and 12 µl of RNA-free water at 70 °C for 5 min. After this denaturation step, 1.25 mM of each dNTP, 25 units of recombinant RNasin® Ribonuclease Inhibitor, and 200 units of M-MLV RT were added and the cDNA synthesis carried out at 37 °C for 60 min. The cDNA products were amplified using the same conditions as described for DNA.

### Barcoding and sequencing

The PCR products generated from one DNA sample (i.e., individual mosquito) were then pooled together: the products from all parasite and viral amplifications were pooled at equal molar concentration (pool1) and the products of the remaining amplifications were pooled together (pool2) in various proportions reflecting the sequence diversity expected at each locus (75% Bacteria_16S rRNA, 5% S200X6, 5% CulicCox1, 10% KDR, 5% mammalian_16S rRNA). Of these, 384 pools (i.e., products from a single mosquito or water control) were then randomly assigned to a well of a 384-well plate and reamplified in a second PCR to add a unique barcode and the Illumina sequencing adaptors [34]. Finally, the barcoded product pools from all mosquitoes were combined (pool1:pool2, 1:3 ratio) and sequenced simultaneously on Illumina HiSeq 2500 sequencing platform (Illumina Inc., San Diego, CA, USA) using a protocol allowing the generation of 300-bp paired-end reads [35].

### Bioinformatic analyses

For the bioinformatic analyses, we first used the sequences of the barcodes incorporated in the second PCR to assign each read to an individual sample. Next, we used the first 18–27 nucleotides of each read to identify the sequence of the PCR primers used to amplify a given sequence and separated the reads by loci. Further analyses were performed for each locus separately as indicated below.

#### Microbiota assessment

All reads carrying bacterial 16S rRNA primers (see above) were analyzed in DADA2 [36] (v1.6.0) by first trimming low-quality bases at the end of each read pair (> 250 bp for forward reads, > 210 for reverse reads) using the following parameters: *maxN* = *0*, *maxEE* = *2*, *truncQ* = *2*. Dereplication was done by combining identical reads and assigning the number of reads belonging to each unique read (*derepFastq*). Next, the dereplicated data were analyzed with the *dada* core sample inference algorithm followed by the merging of read pairs that overlap by at least 12 bases (*mergePairs*). An amplicon sequence variant table (ASV) table was constructed with *makeSequenceTable* for all samples, and chimeras were removed using *removeBimeraDenovo*. For taxonomic assignment, Silva (v128) [37] was used as a training set (using *assignTaxonomy* and *addSpecies*) to create taxonomy data. Finally, the R (v3.4.0) package phyloseq (v1.25.2) [38] was used to combine the ASV table, taxonomy data, and sample metadata for downstream microbiome data analyses. Samples with < 5000 reads were discarded from further analysis, as they likely represent low-level cross contamination. Principal coordinates analysis (PCoA) with Bray-Curtis dissimilarity and weighted UniFrac distance matrices were calculated in phyloseq. Finally, analyses using the Adonis function were performed to simultaneously evaluate the contribution of each factor to the bacterial composition: this multivariate analysis provided a statistical assessment of the association of each factor (i.e., collection site, mosquito species, *kdr*-w genotype, blood-meal status, and infection status; see following sections for details) with bacterial diversity, as well as an estimate of the proportion of the variance explained.

#### Eukaryotic parasites, viruses, and blood-meal composition assessment

First, reads that did not contain the exact barcode and primer sequences were discarded and the remaining reads were assigned to a given sample according to their unique barcode sequence. In order to remove low-quality bases and sequences that were likely primer dimers, each sequence was searched for the forward and reverse primers and trimmed after the reverse primer (if both primers are found). Those sequences where the forward primer was found but the reverse primer was missing were left untrimmed. Untrimmed sequences that belonged to a primer with an expected amplicon length of < 300 bp were trimmed from 50 bp from the 5′ end for further quality filtering. Afterwards, filtered paired-end sequences from each read pair were merged using FLASH [39] to generate a consensus sequence of the overlapping region. Each correctly merged sequence was trimmed of both amplification primers (forward and reverse) and kept only if the sequence was longer than 90 bp. Then all concatenated sequences amplified with the same primer pair (from all samples) were compared to each other, and a single copy of each unique sequence was kept (while the number of times it was observed in each sample was recorded). Unique sequences that were observed less than ten times across all samples were removed as they likely represent instances of sequences carrying errors [34, 40]. The remaining unique sequences were compared against all DNA sequences annotated on the NCBI nr database using BLAST [34]. We then retrieved the taxonomic information of the most similar sequence(s) if there was at least 70% identity over the entire sequence length. Finally, we summarized the parasite and virus species identified, the percentage identity (i.e., similarity to the most similar sequence[s] on NCBI), and the number of reads observed in each sample.

When evaluating the blood-meal composition of each mosquito, the same procedure as just described was applied, but only samples with at least 1000 reads were considered (to avoid including possible cross-contamination or sequences mis-assigned to one sample due sequencing errors in the barcode) [41]. The distribution of reads generated from the mammalian_16S rRNA primers for all mosquito and negative control samples is presented in Additional file 3: Figure S2.

#### *kdr* genotype (L1014F) and species determination (S200X6.1 and COX1)

Reads amplified from the *kdr* and S200X6.1 primer pairs were processed as described above, with sequences assigned to their specific sample, filtered for quality, and merged with FLASH.

For the L1014F locus, the top two most abundant unique sequences in each sample (Seq1 and Seq2) were considered for further analysis. If one sample is homozygous, the second most abundant sequence will be a read carrying sequencing error(s) and should account for a small fraction of the Seq1 reads (i.e., Seq2/[Seq1+Seq2] ~ 0). Alternatively, if one sample is heterozygous, we would expect the number of Seq2 reads to be very close to Seq1 and Seq2/(Seq1+Seq2) ~ 0.5. We calculated the ratio Seq2/(Seq1+Seq2) for all samples and, based on the distribution (Additional file 4: Figure S3) determined cut-offs for homozygous and heterozygous genotypes (taking into account small deviation from expectation due to sequencing errors). Only samples with at least 1000 reads were considered for further analysis.

For the S200X6.1 locus [33], the most abundant sequences for each sample were compared against DNA sequences on the NCBI nr database as described above. For samples with ≥ 1000 reads, the species level taxonomy was retrieved and the *Anopheles* species as well as the total read count were summarized. Sequences generated from the primers targeting the COX1 gene (CulicCox1) were used to identify *An. nili* species in samples as this species is not successfully amplified with the S200X6.1 primers [33, 42].

## Results

### Bacterial composition of *Anopheles* mosquitoes from West Africa

**Fig. 1:**
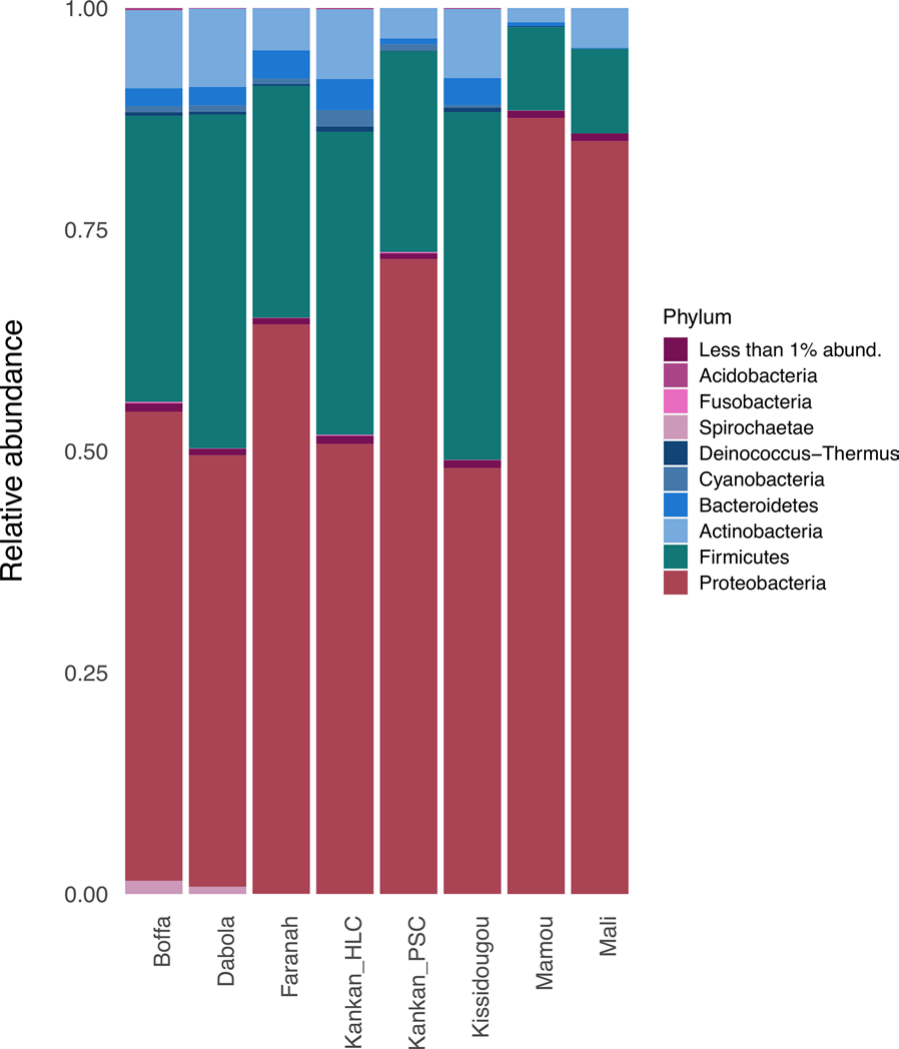
Average relative abundance of bacterial phyla from each mosquito collection site in Guinea and Mali. Bacterial species from the *Proteobacteria* phylum are the most abundant, followed by *Firmicutes* and *Actinobacteria*.* Less than 1% abund.* represents the aggregate of all phyla that make up < 1% of all bacteria

DNA was extracted from 665 individual *Anopheles* mosquitoes collected in Guinea (*n* = 584) and Mali (*n* = 81). To characterize the microbiota of each mosquito, we amplified and sequenced the V2 variable region of the bacterial 16S ribosomal RNA genes (see Methods for details). We obtained a total of 8,467,703 sequences derived from 760 samples (665 mosquitoes and 95 extraction controls). On average, each mosquito sample yielded 11,730 sequences (minimum = 259, maximum = 29,908) compared to 5984 sequences on average per extraction control (minimum = 31, maximum = 15,946). We assigned these sequences to 21,527 ASVs (analogue of operational taxonomic units [36]), representing 37 phyla, including *Proteobacteria* (6692 ASVs accounting for 64% of all reads), *Firmicutes* (26%), *Actinobacteria* (6%), and *Bacteroidetes* (2%) (Fig. 1; Additional file 5: Figure S4).

**Fig. 2:**
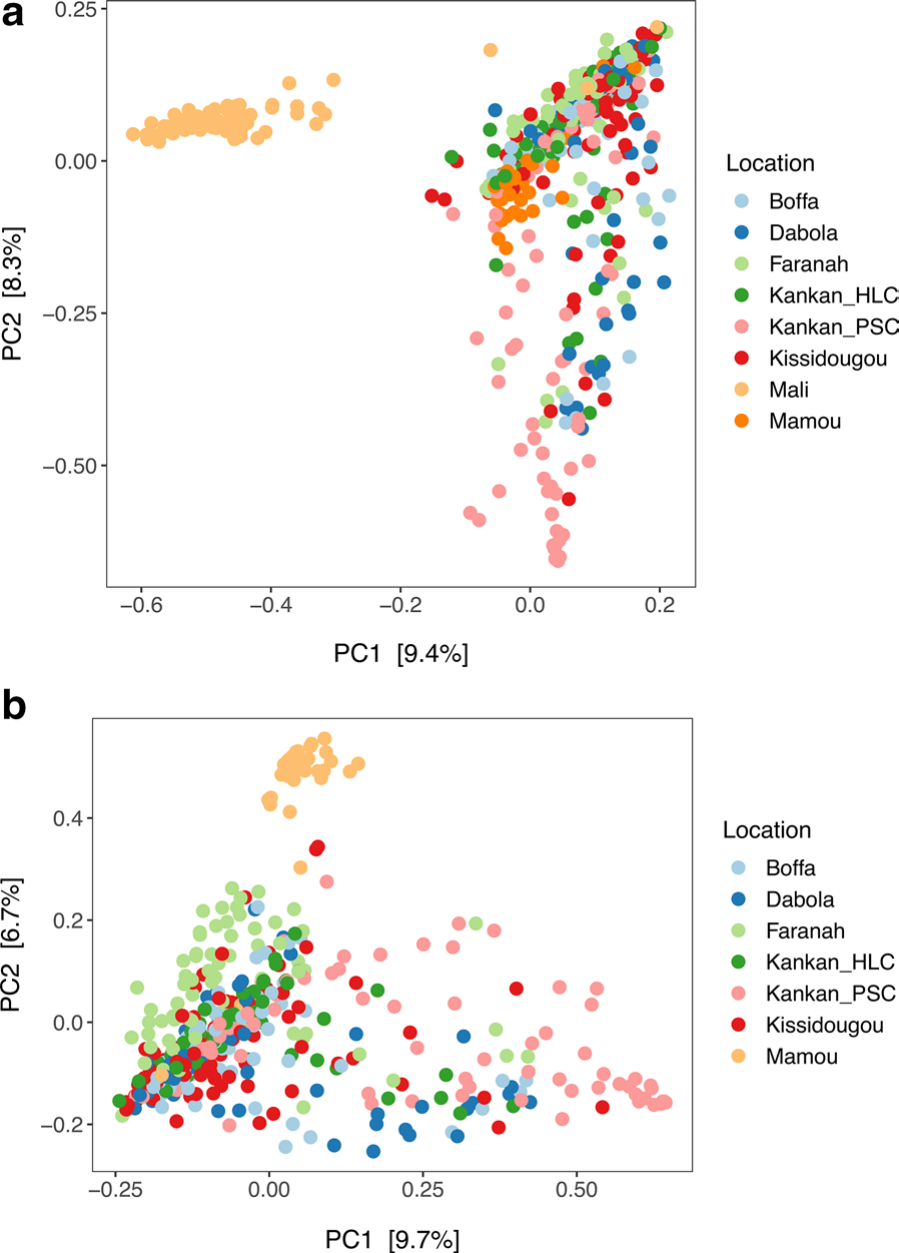
Principal coordinates analysis plot showing the dissimilarity between the microbial composition of individual mosquitoes based on the Bray-Curtis dissimilarity metric for sites in Guinea and Mali (**a**) and Guinea only (**b**). Each dot represents the bacterial composition of a single mosquito. The numbers in brackets near the axes indicate the proportion of the variance explained by the principal components 1 and 2 (*PC1*,* PC2*, respectively)

To investigate differences in bacterial composition among mosquitoes, we calculated β-diversity estimates using weighted UniFrac and Bray-Curtis dissimilarity matrices. PCoA performed using Bray-Curtis dissimilarity or weighted UniFrac distances showed that the microbial composition separates mosquitoes into distinct clusters (Fig. 2a; Additional file 6: Figure S5a). These clusters appeared to group mosquitoes collected in the same sites (Fig. 2), and this observation held true when we restricted our analyses to mosquitoes only collected from sites in Guinea (Fig. 2b; Additional file 6: Figure S5b). The details of all the ASVs identified and their taxonomy are provided in Additional file 7: Table S2.

### Assessment of mosquito species and *kdr* mutation

We simultaneously genotyped the same mosquitoes at loci informative of their species and insecticide resistance status by high-throughput sequencing (see Methods).

**Fig. 3:**
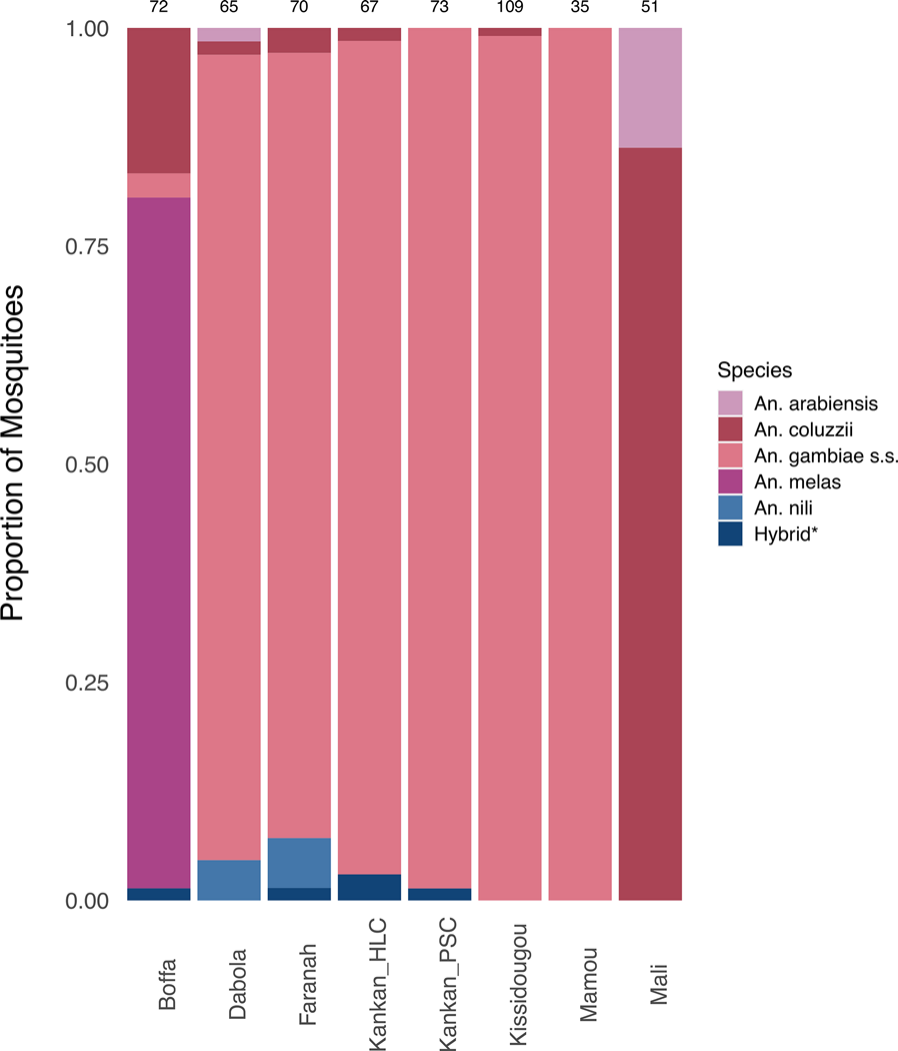
Mosquito species diversity across collection sites in Guinea and Mali. Hybrid* represents samples identified as heterozygous for *Anopheles gambiae* and *An. coluzzii* at the S200X6.1 locus. Numbers above each bar represent the total number of mosquitoes with successfully characterized species from each site

Of 665 mosquitoes, 551 (82.9%) were successfully genotyped for the S200X6.1 [33] and *cox*1 [42] loci. We primarily used the S200X6.1 locus to identify the species of each mosquito as this locus: (i) was more robustly amplified and sequenced than the *cox1* locus (with an average read count of 2917 and 1181 per mosquito, respectively) and (ii) provided clearer taxonomic resolution (with, for example, 233–234 [mean 233.67] nucleotides differentiating the sequences from *Anopheles gambiae* (*s.s.*) from those of *Anopheles coluzzii*, compared to 0–4 [mean 1.71] nucleotide differences using the *cox*1 locus) (Additional file 8: Table S3). However, the S200X6.1 locus systematically failed to yield sequences for some mosquitoes that were identified at *Anopheles nili* using the* cox*1 sequences. Overall, we identified that the mosquitoes belonged to five *Anopheles* species: 404 mosquitoes (74.5%) were identified as *An. gambiae* (*s.s.*), while the remaining mosquitoes consisted of *An. coluzzii* (61 mosquitoes, 11.3%), *An. melas* (57 mosquitoes, 10.5%), *An. arabiensis* (8 mosquitoes, 1.5%), and *An. nili* (7 mosquitoes, 1.3%) (Fig. 3). We also identified five mosquitoes that were heterozygous for the S200X6.1 locus and likely represented F1 hybrids of *An. gambiae* (*s.s.*) and *An. coluzzii* species. The species distribution varied extensively between locations, with *An. gambiae* (*s.s.*) accounting > 90.00% of all mosquitoes collected in five of six locations in Guinea, while *An. melas* was the most abundant species (79.2%, 57/72) in Boffa, a coastal region in western Guinea, and *An. coluzzii* predominated in Bandiagara, Mali (86.3%, 44/51) (Fig. 3).

**Fig. 4:**
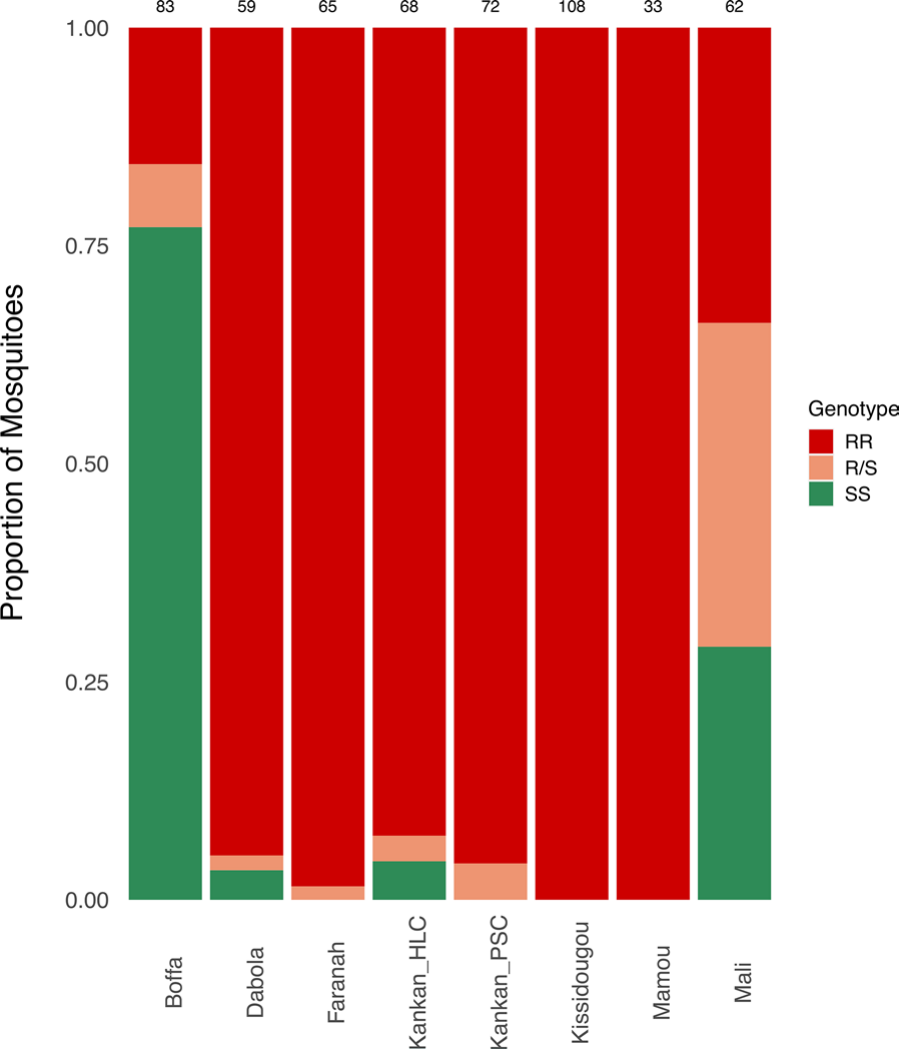
Distribution of mosquito knockdown resistance west (*kdr*-w [L1014F mutation variant]) in mosquitoes collected across Guinea and Mali. Numbers above each bar represent the total number of mosquitoes successfully genotyped at the *kdr*-w genotype, per site. *RR* Homozygous resistant, *SS* homozygous sensitive, *R/S* heterozygous

Pyrethroid resistance is often due to a point mutation in the voltage gated sodium channel gene, described as knockdown resistance (*kdr*) [32]. 550 (82.7%) of the mosquitoes were successfully genotyped at this locus (with an average coverage of 2,436 reads per mosquito). In Guinea, with the exception of mosquitoes collected in Boffa, most mosquitoes (>92.6%) were homozygous for the *kdr*-w (L1014F) alleles that is associated with resistance to pyrethroids [32] (Fig. 4). In Boffa, where *An*. *melas* is the predominant species, most mosquitoes were homozygous for the wild-type allele (L1014L). In Mali, the distribution was more heterogeneous, with roughly equal proportions of mosquitoes homozygous for the wild-type, resistant allele or heterozygous. Across mosquitoes, the genotype at the *kdr*-w locus correlated almost perfectly with the mosquito species, with *An. gambiae* carrying primarily L1014F alleles while *An. arabiensis* and *An. melas* were essentially wild-type. Only *An. coluzzii* showed high proportion of both alleles (Additional file 9: Figure S6). The details of all the genotypes and sequences amplified from each mosquito are provided in Additional file 10: Table S4.

### Determination of the blood-meal composition

**Figure 5:**
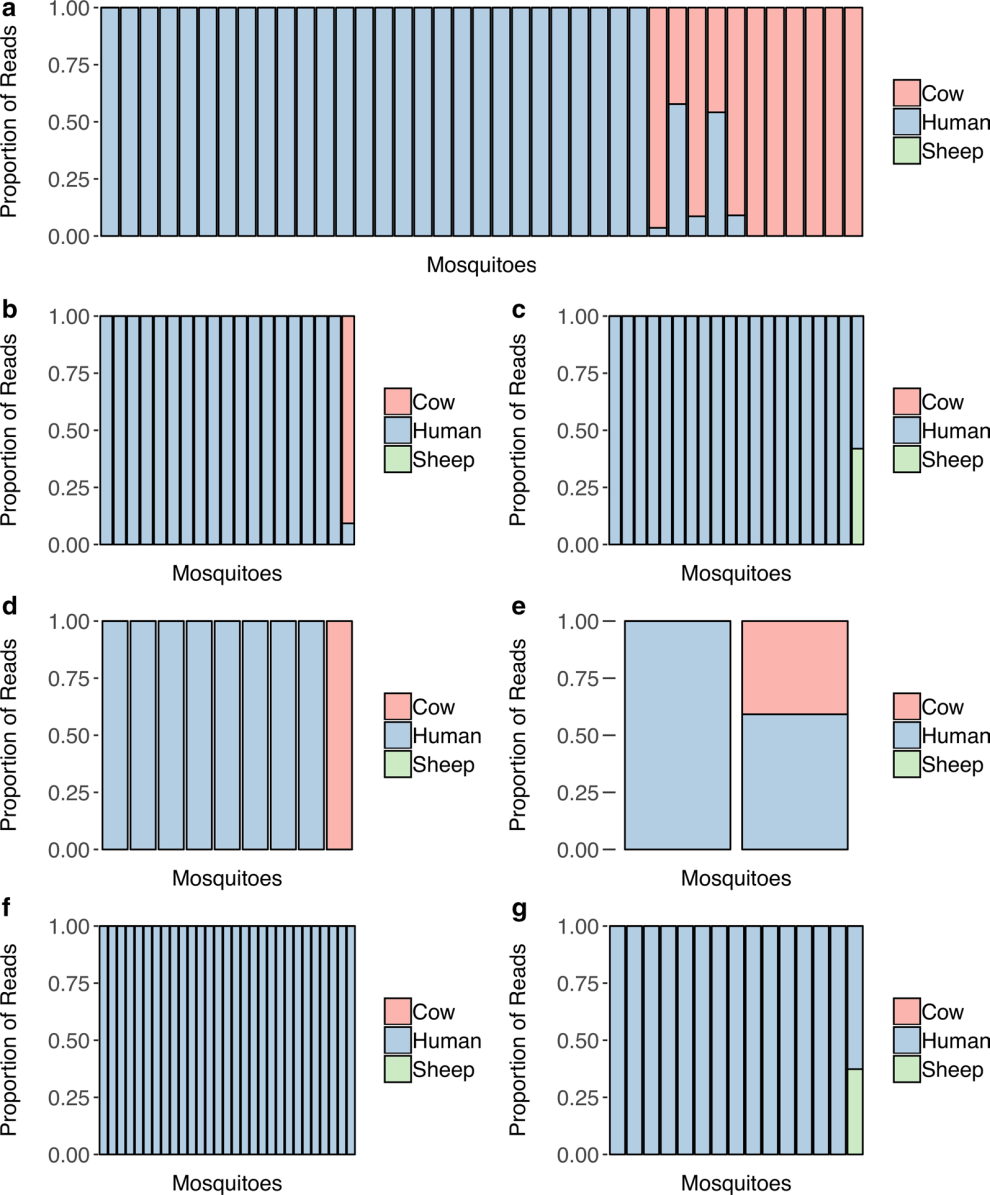
Host blood-meal composition of individual mosquitoes collected from Kankan using pyrethrum spray catches (**a**), Kankan using human landing catches (**b**), Kissidougou (**c**), Dabola (**d**), Faranah (**e**), Boffa (**f**), Mali (**g**). Each bar represents an individual mosquito

To characterize the composition of the last blood meal of each of these mosquitoes, we used the same DNA extract to amplify and sequence a short fragment of the mammalian mitochondrial 16S rRNA gene. Of these, 133 mosquitoes yielded > 1000 reads and were considered blood fed in later analyses. A total of 126 mosquitoes carried human DNA, 14 carried cow DNA, and two carried sheep DNA (Fig. 5; Additional file 11: Table S5). Nine mosquitoes fed on more than one mammalian host species (Fig. 5). The blood-meal composition differed between sites with, for example, 12 mosquitoes (20.1%) from Kankan that fed, at least partially, on cow blood while mosquitoes from all other sites in both Mali and Guinea fed almost exclusively on human blood.

### Identification of eukaryotic parasites and viruses from individual mosquitoes

A sequencing-based assay recently developed in our laboratory [34] was then used to determine whether each mosquito carried a eukaryotic parasite and/or arbovirus. After PCR amplification and subsequent sequencing of DNA extracted from individual mosquitoes, we identified DNA sequences from eukaryotes and viruses from 127 mosquitoes, with an average of 1876 reads supporting each identification in each mosquito. Of the 95 extraction controls (water samples that were processed simultaneously), nine were also positive for one or more parasites, but with an average of 54 reads per parasite (Additional file 12: Table S6). The low read counts in those water controls could be explained by low-level cross-contamination during extraction or by sequence mis-assignment due to sequencing errors in the Illumina index sequences [34].

Eight mosquitoes (1.2%) from three sites, six of which were identified as *An. gambiae*, yielded DNA sequences identical to *Plasmodium falciparum*, the primary cause of human malaria in Africa. We detected DNA belonging to *Theileria* species in a relatively high number of mosquitoes (27/665, 4.1%). *Theileria* species are protozoan parasites that can be infective to domestic (i.e., cattle) and wild (buffalo) animals and are transmitted by ticks [43]. Interestingly, from the 14 samples for which *Bos indicus* (cow) was identified with > 500 reads, *Theileria* species were detected in 12 (86%), suggesting the tick-transmitted parasite may have been ingested by these mosquitoes during their last blood meal. Seven mosquitoes yielded a DNA sequence identical to *Loa loa* and to several other filarial worms (and were further characterized as deriving from *Mansonella perstans* after sequencing of longer amplification products; M. Cannon, personal communication). Several DNA sequences were closely related to known parasites of mosquitoes themselves, belonging to microsporidia [44] (e.g., *Parathelohania *sp.), mosquito-transmitted nematodes (e.g., *Setaria *sp. [45, 46]), as well as two recently discovered *Anopheles* flaviviruses (e.g., *An. flavivirus* variant 1 and variant 2) [47] (Table 2). The details of all parasite sequences amplified and their taxonomic information for each mosquito are provided in Additional file 12: Table S6.

**Table 2:** Eukaryotic parasites and viruses identified from screening mosquito samples

Taxon targeted	Species identified (*N* positive)	Percentage Positive	Percentage Identity
Apicomplexa A	*Theileria *sp. (24)	3.60	100
Apicomplexa B	*Plasmodium falciparum* (8)	1.20	100
Apicomplexa C	*Theileria *sp. (3)	0.45	100
Microsporidia	*Parathelohania anopheles* (38)	5.71	92.47
	*Hazardia milleri* (1)	0.15	97.38
	*Culicospora magna* (6)	0.90	99.7
	*Microsporidium *sp. 3 NR-2013 (34)	5.11	97.01
Nematoda A	*Acanthocheilonema viteae* (12)	1.80	100
	*Loa loa*/*Dipetolenma *sp. (7)	1.05	99.64
	*Setaria labiatopapillosa* (11)	1.65	100
	*Auanema rhodensis* (1)	0.15	98.21
Nematoda B	*Setaria yehi*/*Setaria digitate* (4)	0.60	99.72
	*Acanthocheilonema viteae* (1)	0.15	99.72
	*Dipetolenma* sp./*Filariodea* sp. (3)	0.45	98.94
Flavivirus	*Anopheles flavivirus* variant 2 (2)	0.30	99.06
	*Anopheles flavivirus* variant 2/variant 1 (1)	0.15	88.26
	*Culex flavivirus* (1)	0.15	99.06

Table shows the parasite and viral taxon targeted by each primer, the species identified and number of mosquitoes positive for that species, the percentage of total mosquitoes positive, and the percentage match of the sequences amplified compared to that of the NCBI database

### Evaluation of the factors influencing microbial composition of wild mosquitoes

The characterization of the mosquito species, insecticide resistance associated genotype, blood-meal status, and infection from the same mosquitoes that have been examined for their microbial diversity enables a rigorous assessment of the relative contribution of these factors to the microbial composition. Note that to avoid possible biases introduced by sample storage or DNA extraction, we restricted this analysis to mosquitoes collected in Guinea that were all processed identically and simultaneously. We implemented an analysis of variance [48] to simultaneously test the contribution of each factor, while accounting for the others (multivariate analysis). The geographical location of the samples explained most of the variation in microbial composition (*R*^2^ = 0.200, *ρ* = 0.001), whereas the mosquito species (*R*^2^ = 0.004, *ρ* = 0.208), *kdr-*w genotype (*R*^2^ = 0.006 *ρ* = 0.438), and feeding status (*R*^2^ = 0.003, *ρ* = 0.173) were not statistically associated with the bacterial composition. The mosquito infection status was significantly associated with the microbial composition but had a very marginal effect (*R*^2^ = 0.015, *ρ* = 0.001) (Table 3). To further investigate the relative roles of geography and species that are partially confounded in this dataset, we examined PCoAs of the bacteria composition, restricting the analysis to (i) all *An. gambiae* collected in seven sites (Additional file 13: Figure S7a) and, separately, (ii) mosquitoes from all *Anopheles* species collected in Boffa (Additional file 13: Figure S7b). Together, these analyses validated the results of the statistical testing and confirmed that geographical location of the mosquitoes had a much greater influence on the bacterial composition than did the species of the mosquitoes.

**Table 3:** Relative contribution of mosquito factors to the microbial composition

Factor	*df*	*R*^2^	*P* value
Location	7	0.2	0.001
Mosquito species	4	0.004	0.208
*kdr-*w genotype	2	0.006	0.438
Blood meal	1	0.003	0.173
Infection status	1	0.015	0.001
Residuals	440	0.772	

Table shows, for each factor, the *df*, *R*^2^ (percentage of variation explained), and *P* value (significance value) calculated using the Adonis function

*df* Degrees of freedom;* kdr*-w, mosquito knockdown resistance west

## Discussion

The importance of the mosquito microbiota on vector biology and pathogen transmission has been well recognized, with several studies demonstrating the role of endogenous bacteria on the vector’s development [19, 20], immunity [22, 23], and competency [49]. However, few studies have examined the factors that shape the bacterial composition of mosquitoes, and most of those used laboratory-reared mosquitoes to assess influences on bacterial communities [20, 30, 50]. This latter limitation could be especially problematic since bacterial diversity of wild-caught *Anopheles* mosquitoes has been shown to be greater than that of mosquitoes reared in the laboratory [51]. In addition, these studies typically focus on testing the influence of a single factor on the bacterial composition without accounting for confounding factors. In the present study, we examined the microbial diversity of 665 individual wild-caught *Anopheles* mosquitoes collected in six sites in Guinea and one site in Mali. Consistent with previous studies, the bacterial composition of mosquitoes was dominated by bacteria from the phyla *Proteobacteria*, *Firmicutes*, and *Actinobacteria* [26–28]. *Pseudomonas*, a bacterial genus commonly found in mosquito larvae and larval habitats [27], was one of the most abundant genera detected across all samples (Additional file 5: Figure S4; Additional file 7: Table S2). Despite these overall similarities, we observed significant differences in bacterial composition among mosquitoes and examined the contribution of various factors to this diversity. For each mosquito, we characterized their species, *kdr-*w genotype, blood-meal status, and infection with various eukaryotic parasites and viruses. We then simultaneously estimated the relative contribution of each of those endogenous and exogenous factors on the microbial composition of the mosquitoes. In this analysis, the mosquito collection site accounted for approximately 20% of the variation in bacterial composition, whereas the other factors made a marginal or non-significant contribution (Table 3).

Our findings are consistent with previous studies that have shown that collection site is a major contributor to the microbial diversity of field-caught *Anopheles* mosquitoes [26, 52–54]. For example, Muturi et al. found that sampling site has a strong effect on microbial composition and diversity, even in their examination of nine different mosquito species [27]. It should be noted, however, that “collection site” in our study represents a summary of many parameters. In particular, the mosquitoes were collected from very different ecoregions (grasslands and canopy forests, mountainous forests, or savanna, Table 1); as such, the influence of “collection site” on the microbial composition shown in our study could reflect the effect of differences in larval habitats, flora the mosquitoes rely on for nectar feeding, and/or local population differences. In addition, it is worth noting that the bacterial composition of the adult mosquito has been shown to vary depending on the larval breeding sites and the bacterial composition of these aquatic habitats [55]. Also, sugar source appears to have a pronounced influence on the vectoral capacity of *An. sergentii* mosquitoes [56] and has been shown to impact the microbial composition of laboratory-reared adult mosquitoes [57]. Future studies using a denser, more local sampling of wild-caught mosquitoes will be required to better understand the individual contribution of these local parameters.

On the other hand, our analyses provide new insights on the role of factors other than collection site on the microbial composition of mosquitoes. We did not observe any significant contribution of feeding status on microbial variation of the wild mosquitoes. This finding is in contrast to the results reported in a previous study which described that the bacterial diversity of *Aedes aegypti* mosquitoes fed on human, chicken, or rabbit blood was significantly lower than that of newly emerged unfed mosquitoes [30]. This discrepancy could reflect differences between mosquito species/genera or, more likely, differences between wild-caught mosquitoes (that might have had prior blood meals) and laboratory-reared mosquitoes with less variable microbial composition. Similarly, our study did not reveal any significant contribution of genetic factors (i.e., mosquito species, *kdr-*w genotype) on mosquito microbial variation. These observations contrast with those made in a previous study that described distinct bacterial compositions in two species of *Culex* mosquitoes collected from the same site and with an identical larval aquatic environment [28]; it is possible that the different results are due to differences among *Culex* species in their larval feeding habits [58]. In a previous study, the L1014F *kdr* allele frequency was reported as being high or near fixation in the Kankan and Kissidougou sites of Guinea and low in the Boffa site [59], which is consistent with our findings. In theory, insecticide-resistant mosquitoes could display a different microbial composition since this resistance may allow them to survive insecticide exposure that could impact the bacterial populations. In our study, we saw no evidence of the L1014F resistant allele influencing adult mosquito microbial composition, although the lack of information on whether these mosquitoes might have been exposed to insecticides limits the conclusions that can be drawn from this observation. However, given that insecticide resistance alleles in genotypes and mosquito species only represent a small fraction of the genetic factors that could impact the mosquito microbiota and in light of our observation that the collection site is strongly associated with the bacterial composition, it would be interesting to further investigate whether genetic diversity is associated with the microbiota of mosquitoes [60].

Interestingly, we observed a marginal but statistically significant association between infection status (infected *n *= 127* vs* non-infected *n *= 513) and the mosquito microbial composition. Modification of insect gut microbiota by parasitic [61] or viral [62] infections has been demonstrated in a few studies. Pathogenic or non-pathogenic (e.g., insect-specific viruses) species could be involved in crosstalk with insect metabolism pathways or immune system to influence the microbiota [63]. It should be noted that, due to the low infection rate with parasitic and viral species we found in the mosquitoes (< 5.0%), we assessed the influence of infection on the microbiota using an aggregate of all the parasite and viruses we detected (as opposed to individual parasite and virus species), and it is possible that the effect of one organism on the microbiota might be diluted down and undetected once analyzed together with other parasites and viruses that have no influence on the bacterial communities. For example, *Theileria *spp. are transmitted by ticks and unlikely to be viable in mosquitoes and, therefore, they probably contribute little or not at all in terms of influencing infection on the mosquito microbiota. Future studies assessing the direct influence of some of the parasites found in abundance in this study (e.g., *Parathelohania *sp*.*, *Microsporidium *sp.,) and the recently discovered virus (*Anopheles flavivirus*) could further elucidate the tripartite relationship between the mosquito, microbiota, and mosquito-infecting agents. One important caveat of our study is that we screened for RNA virus sequences from DNA extracts and that while this approach successfully detected multiple Flaviviruses, the extraction was not optimized for RNA molecules and many sequences might have been lost, leading to an underestimation of the number of viruses.

Finally, the approach described in this study is easily adaptable to other disease vectors (e.g., ticks and sand flies) or insects important in agriculture (such as bees) and easily customizable to examine specific factors of interest by simply adding or replacing PCR primers.

## Conclusions

In summary, we provide a comprehensive assessment of the microbial composition and diversity of 665 wild mosquitoes and a simultaneous examination of the relative contribution of five different mosquito-related factors on microbial variation. This approach enables rigorous estimation of the importance of these factors to shaping the bacterial composition, while correcting for their often confounding effect. Our results highlight the prominent role of the mosquito collection site and, to a lesser extent, parasitic and viral infection, on shaping the bacterial composition of wild-caught mosquitoes. These findings provide a solid foundation to implement further investigations and examine the specific components of the environment (e.g., bacterial communities of the larval habitats, source of nectar, genetic diversity) shaping the microbial composition of wild mosquitoes and the mechanisms mediating these effects.

## Supplementary information

**Additional file 1**: **Figure S1**: Geographical locations (green pins) of mosquito collection sites in Guinea and Mali. Map image was prepared using the online ArcGIS® software by ESRI.

**Additional file 2**: **Table S1**: Summary of all primers used in the study. Table shows, for each primer pair, the loci targeted, the base pair length of amplicon, and the forward and reverse primer sequences.

**Additional file 3**: **Figure S2**: Distribution of reads counts for the mammalia_16S primer across mosquito samples and negative control samples.

**Additional file 4**: **Figure S3**: Distribution of the Seq2/(Seq1+Seq2) ratio across samples with one or more reads for the KDR primer used to determine genotype for the *kdr* locus. Samples with a ratio < 0.15 (left dashed line), between 0.15 and 0.35 (between dashed lines), and > 0.35 (right dashed line) were deemed homozygous, non-called, and heterozygous, respectively

**Additional file 5**: **Figure S4**: Average relative abundance of bacteria at the family (**A**) and genus (**B**) level in terms of taxonomic classifications from each mosquito collection site in Guinea and Mali.* Less than 2% abund.* Phyla that make up < 2% of all bacteria

**Additional file 6**: **Figure S5**: PCoA plot showing the dissimilarity between the microbial composition of individual mosquitoes based on weighted UniFrac metric for sites in Guinea and Mali (**A**) and Guinea only (**B**). Each dot represents the bacterial composition of a single mosquito. The numbers in brackets near the axes indicate the proportion of the variance explained by the components 1 and 2

**Additional file 7**: **Table S2**: ASV taxonomy. Table shows, for each ASV, the sequence identified, the taxonomic information (Kingdom to Species), and abundance values. NA represents when an ASV is unknown at that taxonomic rank

**Additional file 8**: **Table S3**: Pairwise comparison of* cox*1 and S200X.6 primers for resolution of mosquito species. Table shows, for each pairwise-comparison between two *Anopheles* species, the mean nucleotide difference and range for* cox*1 and S200X.6 loci

**Additional file 9**: **Figure S6**: Distribution of L1014F mutation (*kdr-*w) in mosquitoes grouped by *Anopheles* species. Numbers above each bar represent the total number of mosquitoes that *kdr*-w genotype is identified, per site.* RR* Homozygous resistant,* SS* homozygous sensitive,* R/S* heterozygous.

**Additional file 10**: **Table S4**: Summary of species and *kdr*-w determination. Table shows, for each sample, the mosquito collection site, the number of reads belonging to *kdr* alleles and *Anopheles* species and their sequences.* kdr_w* Knockdown resistance west (mutant),* WT* wildtype,* H2O* water controls.* Seq* sequence

**Additional file 11**: **Table S5**: Summary of host blood-meal composition. Table shows, the number of mosquitoes carrying mammalian DNA, the percent match of sequence to the NCBI database, and average count of reads per mammal.

**Additional file 12**: **Table S6**: Eukaryotic parasite and virus identification. Table shows the primer name, sample name, collection site, mosquito species, and taxonomic information per sequence identified. Table also gives, for each sequence, the frequency per sample (count), percentage match to NCBI database (*% Identity*), length of sequence (in bp), and length of the match (in bp) to NCBI sequence

**Additional file 13**: **Figure S7**: PCoA plot showing the dissimilarity between the microbial composition of individual mosquitoes based on Bray-Curtis dissimilarity metric for *An. gambiae* mosquitoes only, from sites in Guinea (**A**) and all *Anopheles* mosquitoes identified in Boffa, Guinea (**B**). Each dot represents the bacterial composition of a single mosquito. The numbers in brackets near the axes indicate the proportion of the variance explained by the PC 1 and 2.

## Data Availability

All data generated or analyzed during this study are included in this published article and its additional files. All raw sequences are available in the NCBI Sequence Read Archive under the BioProject ID PRJNA663576. The custom scripts supporting the conclusions of this article are available in the MosqMicrobiome_Paper repository (https://github.com/Haikelnb/MosqMicrobiome_Paper).

## Abbreviations

ASV: Amplicon sequence variant
DDT: Dichlorodiphenyltrichloroethane
*kdr*-w: Knockdown resistance west
PCoA: Principal coordinates analysis
