# Supplementary figures and images for: Relative contributions of various endogenous and exogenous factors to the mosquito microbiota
Source: Parasit Vectors. 2020 Dec 10;13:619. doi: 10.1186/s13071-020-04491-7 (PMC7726613; doi:10.1186/s13071-020-04491-7)

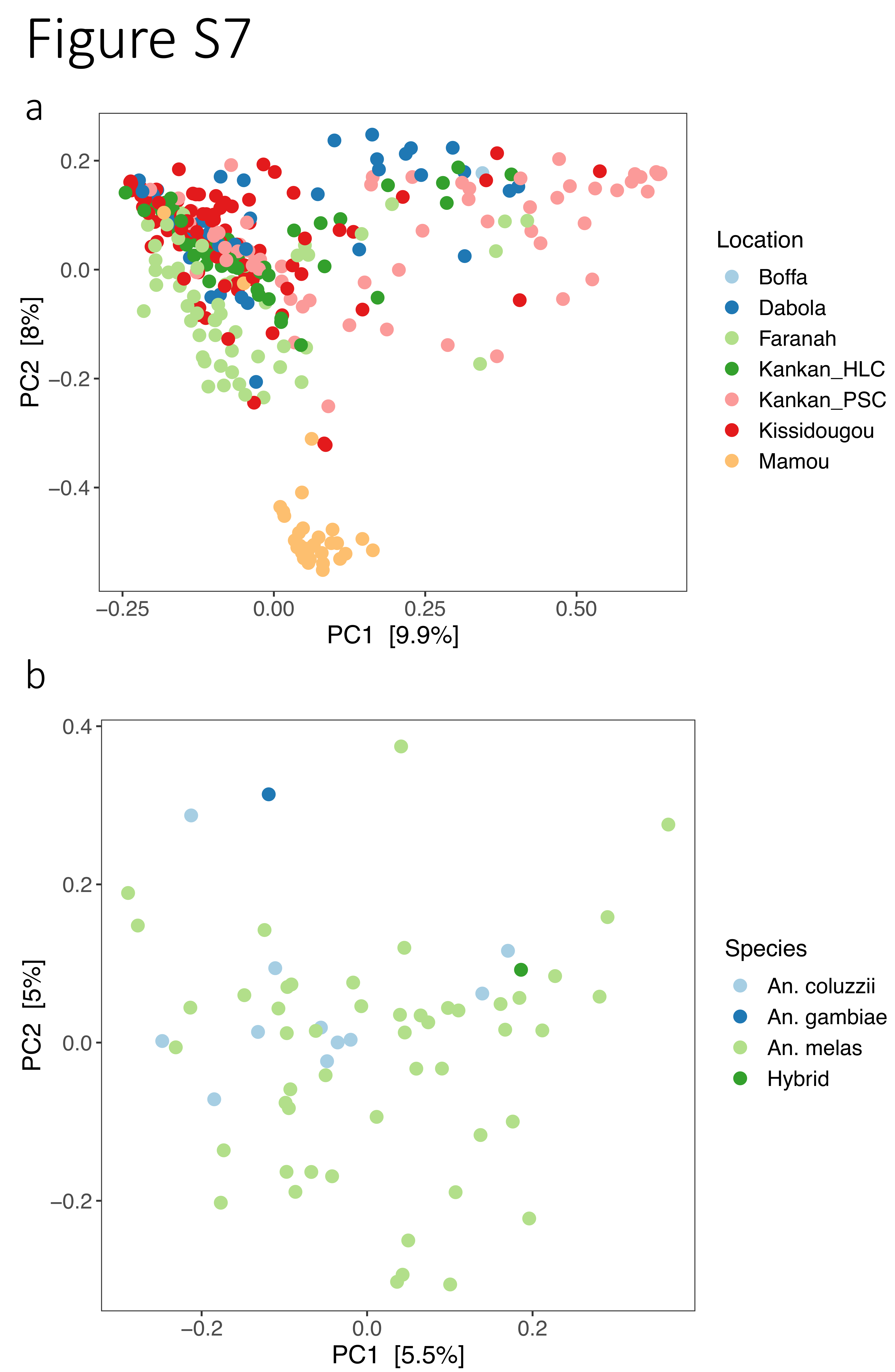

Supplement: Supplementary file 1 — Additional file 1: Figure S1: Geographical locations (green pins) of mosquito collection sites in Guinea and Mali. Map image was prepared using the online ArcGIS® software by ESRI. [file 13071_2020_4491_MOESM1_ESM.tif]

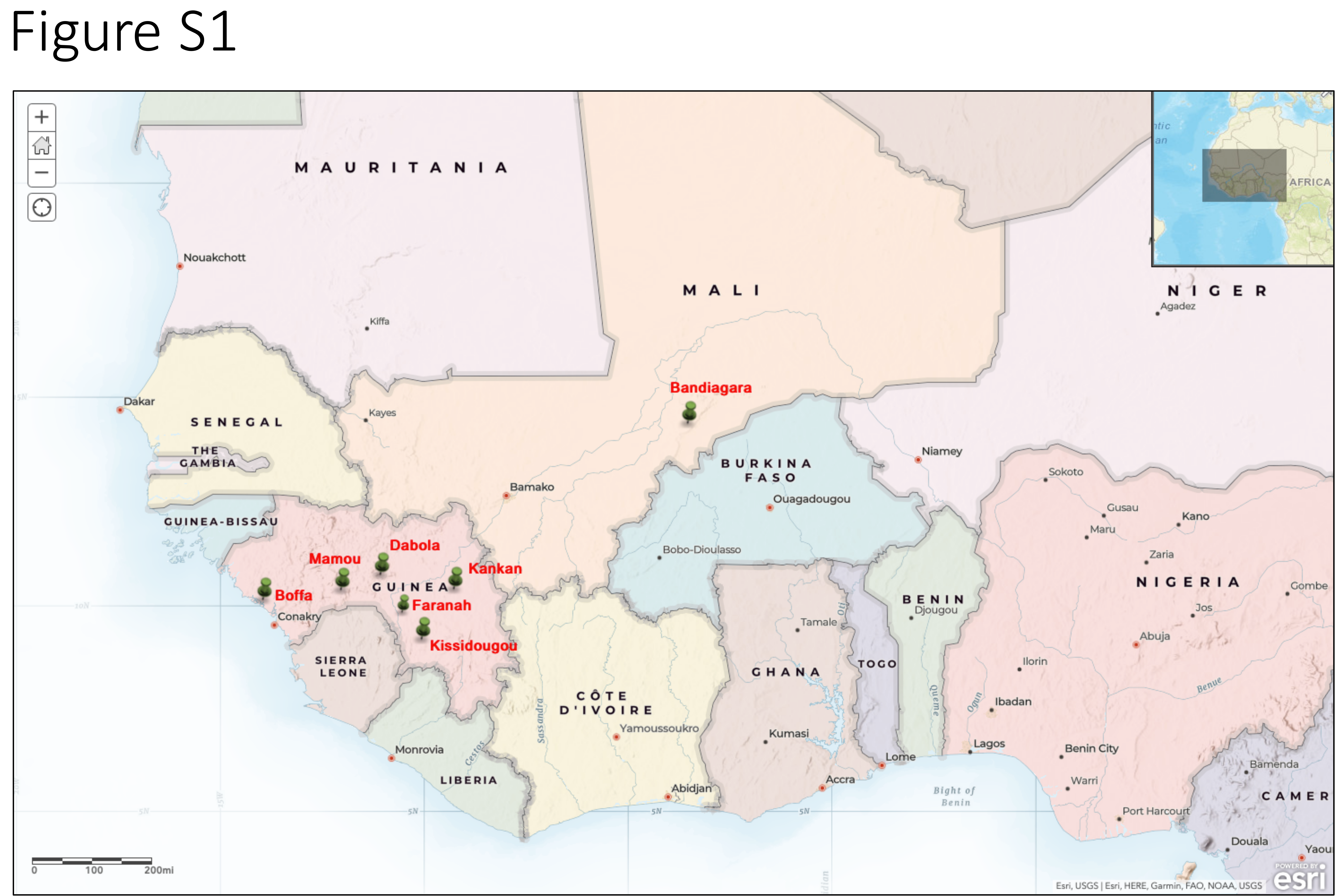

Supplement: Supplementary file 2 — Additional file 2: Table S1: Summary of all primers used in the study. Table shows, for each primer pair, the loci targeted, the base pair length of amplicon, and the forward and reverse primer sequences. [file 13071_2020_4491_MOESM2_ESM.tif]

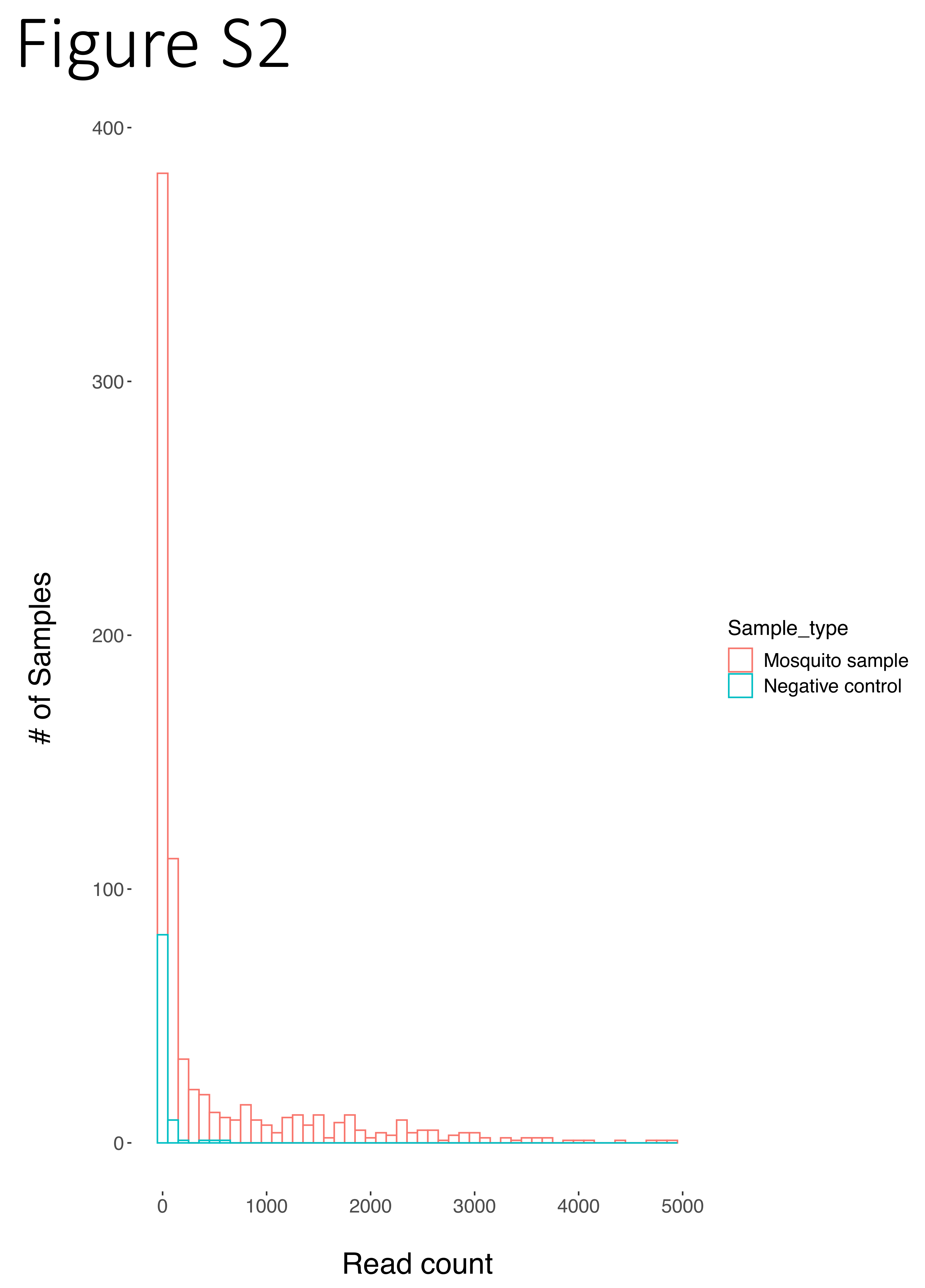

Supplement: Supplementary file 4 — Additional file 4: Figure S3: Distribution of the Seq2/(Seq1+Seq2) ratio across samples with one or more reads for the KDR primer used to determine genotype for the kdr locus. Samples with a ratio < 0.15 (left dashed line), between 0.15 and 0.35 (between dashed lines), and > 0.35 (right dashed line) were deemed homozygous, non-called, and heterozygous, respectively [file 13071_2020_4491_MOESM4_ESM.tif]

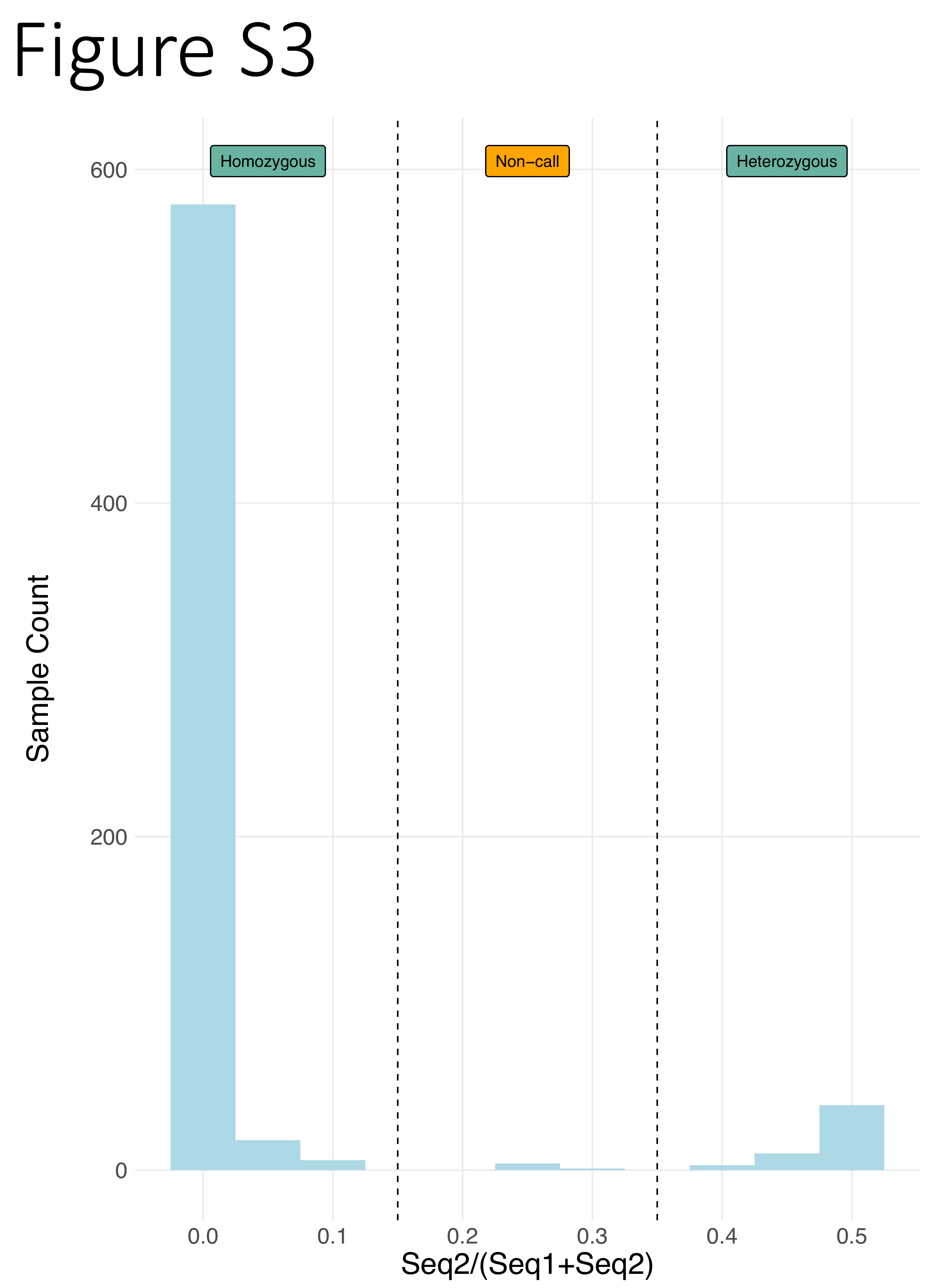

Supplement: Supplementary file 5 — Additional file 5: Figure S4: Average relative abundance of bacteria at the family (A) and genus (B) level in terms of taxonomic classifications from each mosquito collection site in Guinea and Mali. Less than 2% abund. Phyla that make up < 2% of all bacteria [file 13071_2020_4491_MOESM5_ESM.tif]

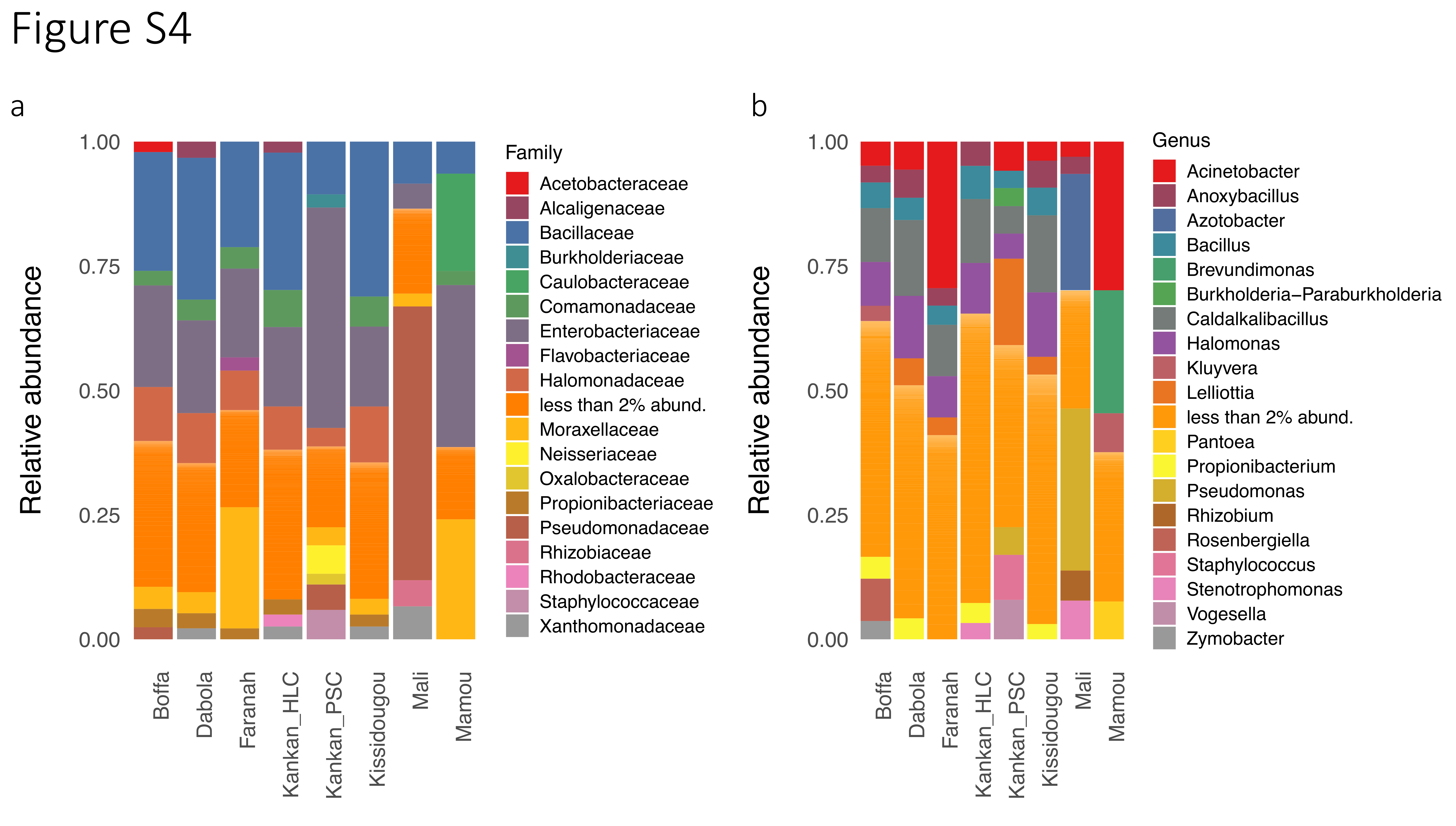

Supplement: Supplementary file 6 — Additional file 6: Figure S5: PCoA plot showing the dissimilarity between the microbial composition of individual mosquitoes based on weighted UniFrac metric for sites in Guinea and Mali (A) and Guinea only (B). Each dot represents the bacterial composition of a single mosquito. The numbers in brackets near the axes indicate the proportion of the variance explained by the components 1 and 2 [file 13071_2020_4491_MOESM6_ESM.tif]

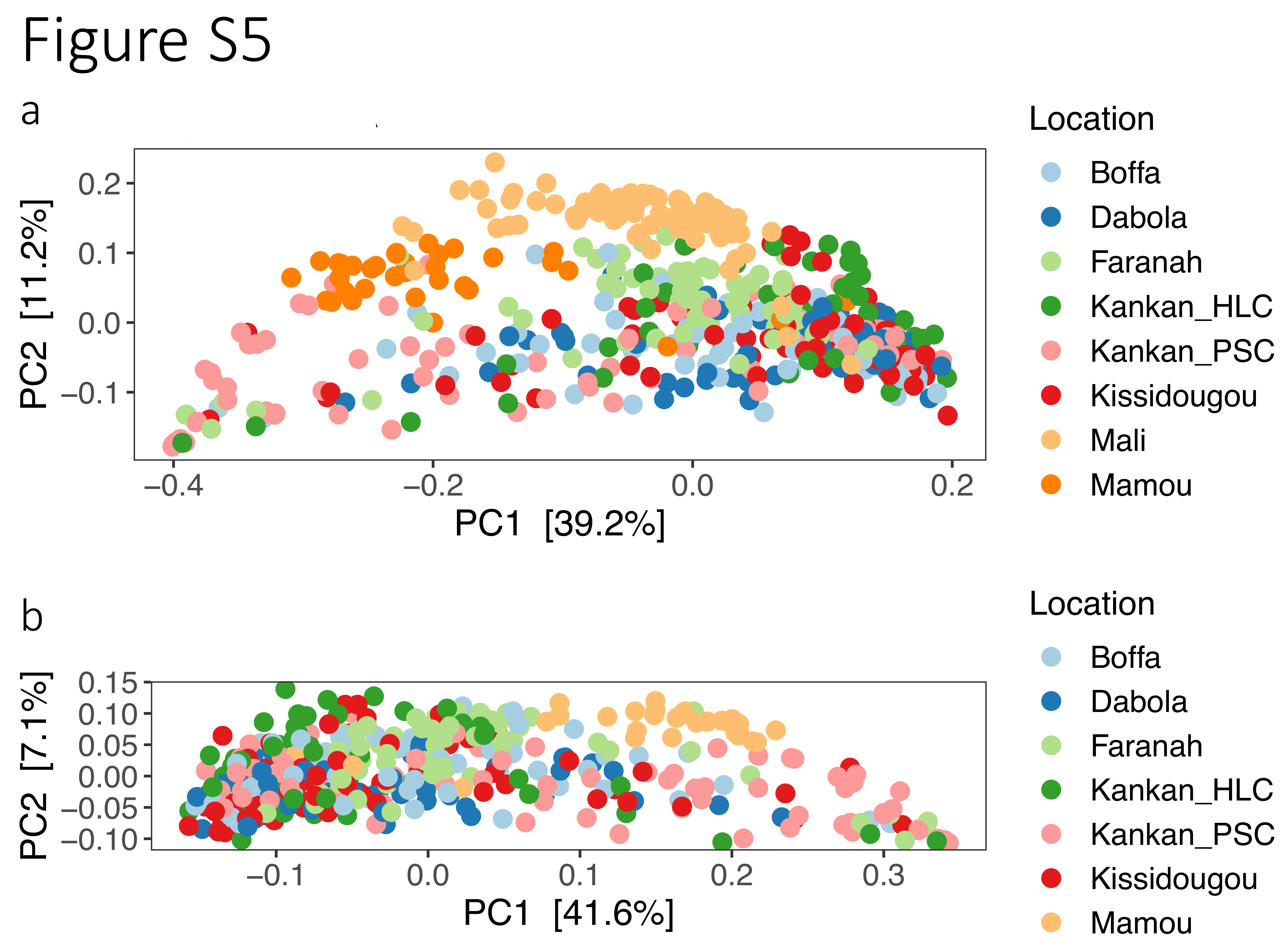

Supplement: Supplementary file 7 — Additional file 7: Table S2: ASV taxonomy. Table shows, for each ASV, the sequence identified, the taxonomic information (Kingdom to Species), and abundance values. NA represents when an ASV is unknown at that taxonomic rank [file 13071_2020_4491_MOESM7_ESM.tif]

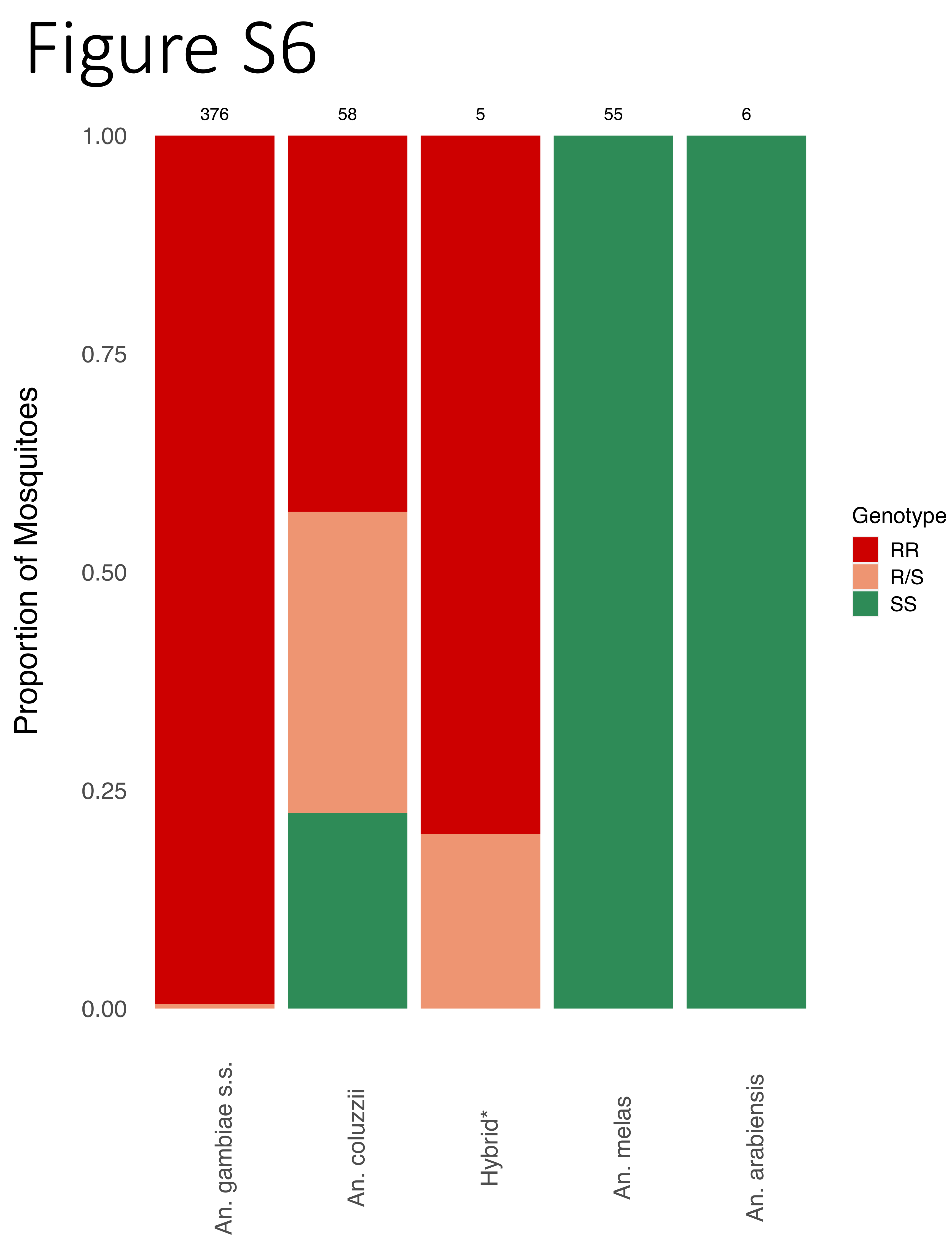

Supplement: Supplementary file 10 — Additional file 10: Table S4: Summary of species and kdr-w determination. Table shows, for each sample, the mosquito collection site, the number of reads belonging to kdr alleles and Anopheles species and their sequences. kdr_w Knockdown resistance west (mutant), WT wildtype, H2O water controls. Seq sequence [file 13071_2020_4491_MOESM10_ESM.tif]
